# The synergized diagnostic value of VTQ with chemokine CXCL13 in lung tumors

**DOI:** 10.3389/fonc.2023.1115485

**Published:** 2023-03-21

**Authors:** Xu Zhang, Yejian Lu, Kenan Huang, Qingfang Pan, Youchao Jia, Baoshuan Cui, Peipei Yin, Jianhui Li, Junping Ju, Xiangyu Fan, Rui Tian

**Affiliations:** ^1^ Department of Ultrasound, Affiliated Hospital of Hebei University, Baoding, Hebei, China; ^2^ Department of Oncology, Hospital of the People’s Liberation Army: 82nd Group Army, Baoding, China; ^3^ Department of Oncology, Affiliated Hospital of Hebei University, Baoding, Hebei, China; ^4^ Department of Radiology, Affiliated Hospital of Hebei University, Baoding, Hebei, China; ^5^ Department of Pathology, Affiliated Hospital of Hebei University, Baoding, Hebei, China

**Keywords:** ARFI, VTQ, lung tumor, malignant pleural effusion, CXCL13

## Abstract

Virtual Touch Tissue Quantification (VTQ) offers several advantages in the diagnosis of various lung diseases. Chemokine expression levels, such as CXCL13, play a vital role in the occurrence and development of tumors and aid in the diagnosis process. The purpose of this study was to evaluate the combined value of VTQ and changes in CXCL13 expression levels for the diagnosis of lung tumors. A total of 60 patients with thoracic nodules and pleural effusion were included, with 30 of them having malignant pleural effusion (based on pathology) and the remaining 30 having benign thoracic nodules and pleural effusion. The relative expression level of CXCL13 was measured in the collected pleural effusions using Enzyme-Linked Immunosorbent Assay (ELISA). The relationship between CXCL13 expression levels and various clinical features was analyzed. A Receiver Operating Characteristic (ROC) curve analysis was conducted on the VTQ results and relative expression levels of CXCL13, and the areas under the curve, critical values, sensitivity, and specificity were calculated. Multivariate analysis incorporating multiple indicators was performed to determine the accuracy of lung tumor diagnosis. The results showed that the expression levels of CXCL13 and VTQ were significantly higher in the lung cancer group compared to the control group (P < 0.05). In the Non-Small Cell Lung Cancer (NSCLC) group, CXCL13 expression levels increased with later TNM staging and poorer tumor differentiation. The expression level of CXCL13 in adenocarcinoma was higher than that in squamous cell carcinoma. The ROC curve analysis revealed that CXCL13 had an area under the curve (AUC) of 0.74 (0.61, 0.86) with an optimal cut-off value of 777.82 pg/ml for diagnosing lung tumors. The ROC curve analysis of VTQ showed an AUC of 0.67 (0.53, 0.82) with a sensitivity of 60.0% and a specificity of 83.3%, and an optimal diagnostic cut-off of 3.33 m/s. The combination of CXCL13 and VTQ for diagnosing thoracic tumors had an AUC of 0.842 (0.74, 0.94), which was significantly higher than either factor alone. The results of the study demonstrate the strong potential of combining VTQ results with chemokine CXCL13 expression levels for lung tumor diagnosis. Additionally, the findings suggest that elevated relative expression of CXCL13 in cases of malignant pleural effusion caused by non-small cell lung cancer may indicate a poor prognosis. This provides promising potential for using CXCL13 as a screening tool and prognostic indicator for patients with advanced lung cancer complicated by malignant pleural effusion.

## Introduction

Lung cancer is one of the leading causes of cancer-related deaths worldwide. According to the World Health Organization, lung cancer accounts for approximately 1.76 million deaths each year. The incidence of lung cancer is higher in men than in women, and it is more commonly diagnosed in older adults. Tobacco use is the leading cause of lung cancer, with long-term exposure to tobacco smoke increasing the risk of developing the disease. Other risk factors for lung cancer include exposure to air pollution, radon, asbestos, and certain genetic mutations. The early detection of lung cancer is crucial for improving survival rates, but due to the lack of symptoms in early stages and the difficulty in diagnosing the disease, the survival rate for lung cancer remains low.

Lung cancer is posing a serious threat to human health, especially in China (1). Unfortunately, patients with early-stage lung cancer often show no distinctive symptoms and are only detected when they reach an advanced stage, leading to a short survival time (1). The gold standard for diagnosing malignant pleural effusion is histopathological examination, but the low probability of finding tumor cells and the high risk of invasive examination for elderly patients with underlying diseases make the diagnosis challenging. In comparison, the detection of tumor markers is a convenient and fast alternative that can not only reflect the occurrence and development of tumors but also assist in diagnosis and prognosis.

Recent research has shown that chemokines play a critical role in the development of tumors (2–7). However, as a single diagnostic indicator, chemokines have limitations in terms of sensitivity and specificity, and false negative and false positive results may occur in some patients. Thus, it is necessary to combine chemokine analysis with other auxiliary examinations for joint diagnosis.

In recent years, lung ultrasound has gained increasing popularity in clinical practice due to the continuous improvement of ultrasound diagnostic equipment. It is easy to use, affordable, reproducible, and does not involve radiation, making it particularly suitable for emergency patients who cannot undergo invasive operations (8–14). Virtual touch tissue quantification (VTQ) has been confirmed to have several advantages in diagnosing various lung diseases.

In light of these developments, this study aims to investigate the diagnostic value of combining VTQ technology and chemokine analysis for lung tumor diagnosis. The researchers hope to find evidence that combining the results of VTQ and chemokine CXCL13 expression could have a high potential value in lung tumor diagnosis. The results also suggest that a higher relative expression of CXCL13 in malignant pleural effusion caused by non-small cell lung cancer may indicate a poor prognosis and could be used as an indicator for screening and prognosis in patients with advanced lung cancer complicated by malignant pleural effusion.

## Materials and methods

### Priori analysis

The study aimed at evaluating the diagnostic accuracy of combining Virtual Touch Tissue Quantification (VTQ) results with chemokine expression levels of CXCL13 in the diagnosis of lung tumors. To ensure that the study was adequately powered to detect the hypothesized effects, *a priori* power analysis was performed. The priori power analysis involved estimating the effect size, determining the sample size required to achieve a desired level of statistical power, and selecting the appropriate statistical test.

The sample size was determined based on the number of patients with malignant pleural effusion (n=30) and the number of patients with benign thoracic nodules and pleural effusion (n=30). The desired level of statistical power was set at 0.8, meaning that the study had an 80% chance of detecting a significant difference between the two groups if such a difference existed. The effect size was estimated based on previous research studies that investigated the diagnostic accuracy of VTQ and CXCL13 expression levels in lung tumor diagnosis.

The statistical test used for the priori power analysis was the two-sample t-test, which is appropriate for comparing the means of two independent groups. The analysis was performed using a statistical software program, such as R or SAS, which allows for the calculation of sample size and power based on the desired effect size, significance level, and level of power.

The results of the priori power analysis determined the sample size required to achieve the desired level of statistical power and provided confidence in the study’s ability to detect a significant difference between the two groups if such a difference existed. By conducting the priori power analysis, the researchers were able to ensure that the study was designed to have sufficient statistical power to detect meaningful differences and reduce the risk of type II errors, or false negatives.

### Research objects

Thirty pleural effusion specimens were collected from newly diagnosed patients with pulmonary malignant tumors and pleural effusion. These patients were admitted to the 82nd Army Group Military Hospital and the Affiliated Hospital of Hebei University from November 2021 to July 2022 and all of the specimens showed the presence of tumor cells. The collection process was performed under sterile conditions, where 10 ml of pleural effusion was collected from each patient. The supernatant was then separated and collected after 10 minutes of centrifugation at 2000 rpm and 4°C. The collected supernatant was stored in -80°C in 1.5 ml eppendorf tubes until further treatment.

The pathological types of the specimens were classified according to the World Health Organization’s histological classification standards for malignant tumors and the staging was performed following the UICC eighth edition TNM staging system. The inclusion criteria for the specimens included the following:

1. Patients’ personal information such as name, gender, age, marital status, race, smoking history, and family disease history
2. Patients who have not received any form of anti-tumor therapy such as radiotherapy, chemotherapy, immunotherapy, or targeted therapy before admission
3. 30 additional specimens, used as the control group, were collected from patients with benign lung tumors and pleural effusion who had no history of malignancy
4. All participants signed an informed consent form approved by the ethics committee of the hospital. The general characteristics of the participants are listed in **Table 1**.

**Table 1:** General characteristics of the participants.

Characteristics		Lung cancer group (n)	Control group (n)
Gender	Male	17 (56.7%)	18 (60.0%)
	Female	13 (43.3%)	12 (40.0%)
Age	≥60	16 (53.3%)	20 (66.7%)
	<60	14 (46.7%)	10 (33.3%)
Smoking history	Yes	21 (70.0%)	17 (56.7%)
	No	9 (30.0%)	13 (43.3%)

There was no significant statistical difference observed in the gender, age, and smoking history of the participants (P>0.05). Specifically, the results showed that the gender had a P-value of 0.79, age had a P-value of 0.29, and smoking history had a P-value of 0.28. The percentage represents the proportion of each characteristic within the group.

### Main instruments and reagents

The following laboratory equipment and supplies were used in this study:

- Siemens ACUSON S2000 Color Ultrasonic Diagnostic Instrument
- US Biotek Microplate Reader (Gen5)
- US Thermo Tabletop Low Temperature High-Speed Centrifuge (CENTRIFUGE PK 121R)
- Thermo -80°C Freezer
- AISITE SPX-150BIII Biochemistry Incubator
- Eppendorf Pipettes
- R&D Systems Inc. Human B-Lymphocyte Chemoattractant (BLC-1/CXCL13) ELISA Kit

### Experimental methods

The Siemens ACUSON S2000 color ultrasonic diagnostic instrument, equipped with VTQ (Virtual Touch Quantification) technology, was utilized in the investigation of lung tumors. A convex array probe was selected for the experiment, and the frequency was set to a range of 3.5-5 MHz. The patients were asked to remain seated in a quiet environment, and the VTQ function was activated to capture the relevant data.

To thoroughly examine the lung tumors, various parameters such as size, margins, internal parenchymal echoes, and blood flow were analyzed using the VTQ technology. A total of five successful measurements were taken, and to reduce the impact of outliers, the maximum and minimum values were removed. The average value of the remaining measurements was then calculated and recorded for statistical analysis.

**Figure 1:**
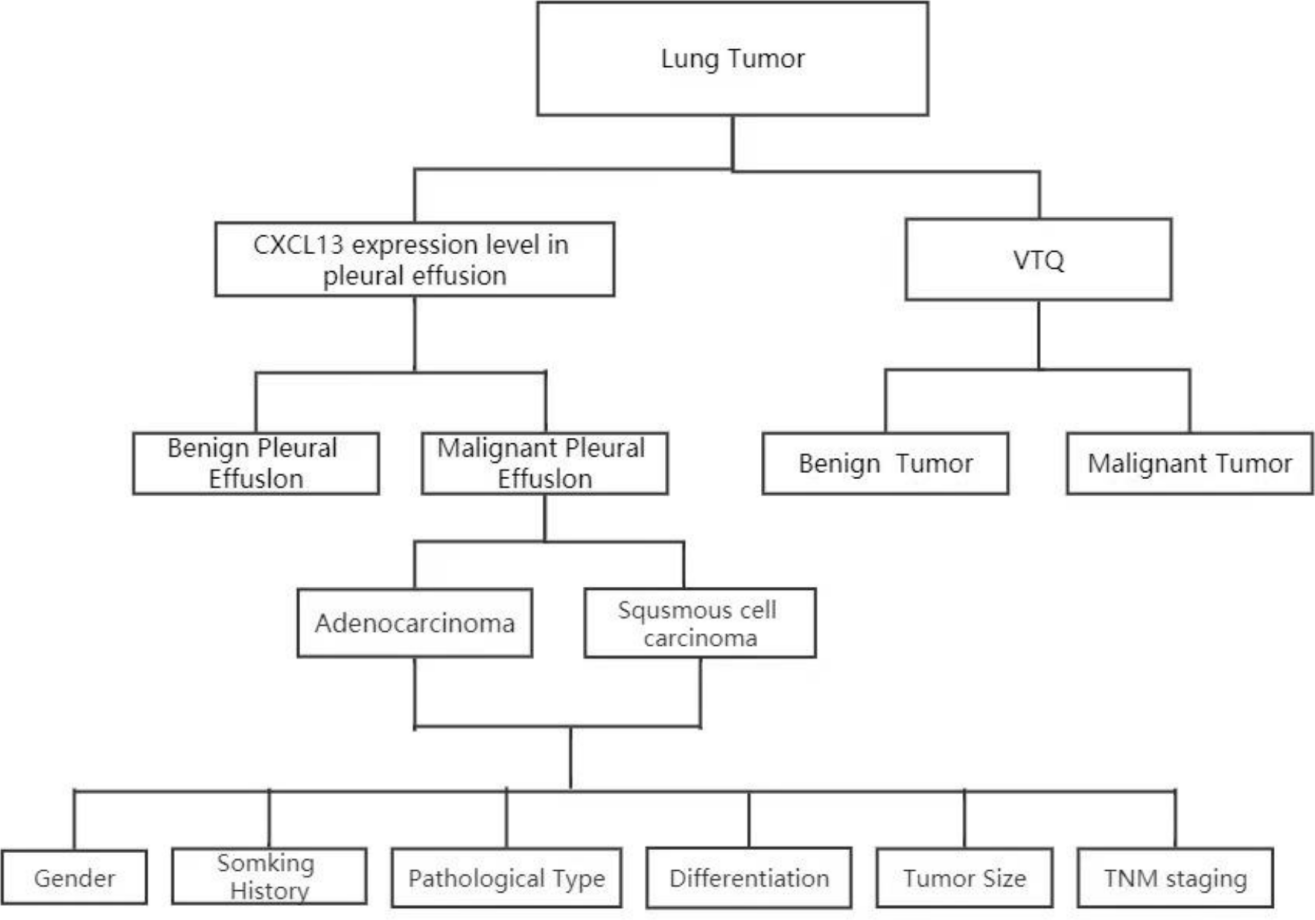
Analysis of CXCL13 and VTQ levels in patients with thoracic tumors and pleural effusion. Patient sample collection: Patients with thoracic tumors and pleural effusion were recruited; CXCL13 concentration measurement: The concentration of CXCL13 in pleural effusion was determined; VTQ measurement: Tissue quantification technology (VTQ) was applied to measure the numerical value of thoracic tumors; CXCL13 and clinical feature analysis: The relationship between CXCL13 and various clinical features was further analyzed; ROC curve analysis using SPSS software: The ROC curve analysis was performed using the multivariate observation values (CXCL13 and VTQ) in SPSS software.

In addition to the VTQ results, the expression level of chemokine CXCL13 was determined through ELISA (Enzyme-Linked Immunosorbent Assay). To obtain an accurate measurement, the pleural effusion specimens were taken out from the -80 oC freezer, thawed to room temperature, and analyzed. The relative expression of chemokine CXCL13 was determined through ELISA, which provides a quantitative measurement of the protein’s concentration in the sample. **Figure 1** summarizes the workflow.

### Statistical methods

The statistical software SPSS19.0 was used to process the data collected in the study. The data was expressed in different ways depending on whether it followed a normal distribution or not. Measurement data that followed a normal distribution were expressed as the mean ± standard deviation ( ± s), while non-normally distributed data were presented as the median (interquartile range).

The non-parametric rank-sum test, also known as the Mann-Whitney Test, was used to compare two independent samples of non-normally distributed measurement data. Unlike the t-test, which assumes that the data follows a normal distribution, the Mann-Whitney Test does not make this assumption, making it a suitable test for comparing two samples of non-normally distributed data.

To examine the relationship between the relative expression level of the chemokine CXCL13 and various clinical characteristics in the non-small cell lung cancer (NSCLC) group, the Mann-Whitney Test was employed. The receiver operating characteristic (ROC) curve was used to determine the optimal diagnostic threshold and corresponding sensitivity and specificity of tumor markers in pleural effusion. The ROC curve plots the true positive rate against the false positive rate for various diagnostic thresholds, allowing for the selection of the threshold that provides the best balance between sensitivity and specificity.

The multivariate ROC was utilized to assess the significance of combined diagnoses based on two or more different indicators. This type of analysis allows for the evaluation of the combined diagnostic power of multiple markers, which may provide a more accurate diagnosis than relying on a single marker alone. A p-value of less than 0.05 was considered statistically significant, meaning that the results observed were unlikely to have occurred by chance.

## Results

### Comparison of relative expression levels of CXCL13 and VTQ results between groups

**Figure 2:**
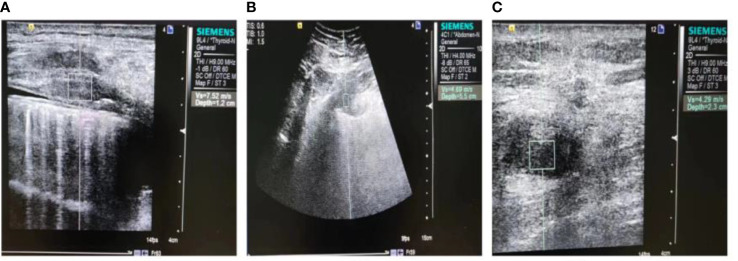
Ultrasound images of malignant chest wall and peripheral non-small cell lung cancers. **(A)** A solid hypoechoic nodule is displayed, with poorly defined borders and an irregular shape. The measurement depth is 1.2 cm and the VTQ value is 7.52 m/s. **(B)** A heterogeneous hypoechoic mass is shown, with unclear boundaries and an irregular shape. The measurement depth is 5.5 cm and the VTQ value is 4.69 m/s. **(C)** Another heterogeneous hypoechoic mass is displayed, with unclear borders, an irregular shape, and a burr-like appearance. The measurement depth is 2.3 cm and the VTQ value is 4.29 m/s.

Our study found that the relative expression levels of CXCL13 and the VTQ values in the lung cancer group were significantly higher compared to those in the control group (P < 0.05). **Figure 2** displays the results of the VTQ measurements.

The differences between the two groups were analyzed and the results are presented in **Table 2**. The table summarizes the mean values, standard deviations, and statistical significance of the differences in CXCL13 and VTQ values between the lung cancer group and the control group. By comparing these values, we can understand the relationship between the expression levels of CXCL13 and the VTQ results, and how they may be used as indicators of lung cancer.

**Table 2:** The CXCL13 expression results and VTQ results from the two groups.

	Lung cancer group	Control Group	P
CXCL13 (pg*/*ml)	879.99 (619.51, 1223.58)	649.12 (525.78, 768.60)	0.002
VTQ (m*/*s)	3.47 (2.14, 4.93)	2.45 (1.99,3.21)	0.02

As the data is non-normally distributed, it is represented by quartiles: the median (50%), the lower quartile (25%), and the upper quartile (75%). The numbers were computed using the SPSS software.

### Investigating the relationship between the relative expression level of CXCL13 and clinical characteristics in NSCLC group

In our study of the Non-Small Cell Lung Cancer (NSCLC) group, we aimed to examine the relationship between the relative expression level of CXCL13, a chemokine, and various clinical characteristics of the patients. To achieve this, we analyzed the expression levels of CXCL13 and compared them to the TNM staging and tumor differentiation of each patient. Our findings indicated that as the TNM staging of the cancer progressed, meaning the cancer became more advanced, and as the tumor differentiation worsened, meaning the cancer cells were becoming more abnormal, the expression level of CXCL13 increased. Furthermore, our results showed that the expression level of CXCL13 was higher in patients diagnosed with adenocarcinoma compared to those with squamous cell carcinoma. These findings suggest that the expression level of CXCL13 may play a role in the progression of NSCLC and can provide valuable information for understanding the underlying mechanisms of this type of cancer. All of these results are presented in **Table 3** for easy reference and further analysis.

**Table 3:** The correlation between the relative expression level of CXCL13 and various clinical characteristics in the NSCLC group.

Characteristics	n	CXCL13 (pg/ml)		
		P50 (P25, P75)	Z	p
Gender				
Male	17	735.11 (610.52, 1225.11)	1.07	0.29
Female	13	960.40 (698.65, 1276.66)		
Age				
*≥* 60	16	720.99 (605.67, 1164.01)	1.54	0.12
*<* 60	14	1016.87 (739.26, 1378.14)		
Smoking history				
Yes	19	777.88 (620.20, 1228.18)	0.37	0.72
No	11	888.14 (617.43,1160.92)		
Pathological type				
squamous cell carcinoma	13	706.86 (588.43,933.24)	2.49*	0.01
adenocarcinoma	17	1160.92 (708.37,1385.22)		
Differentiation				
Low	13	1228.18 (920.91, 1521.25)	3.41*	0.00
Medium and high	17	706.86 (588.43, 882.35)		
Tumor Size				
*≥* 4cm	16	778.88 (605.67, 1323.58)	0.33	0.74
*<* 4cm	14	924.27 (684.50,1166.25)		
TNM staging				
IV a	15	735.11 (600.83, 960.40)	2.43*	0.02
IV b	15	876.82 (636.87,1399.38)		

*Indicates a p-value of less than 0.05. There is a statistical difference with P < 0.05.

### Both the VTQ and CXCL13 biomarkers can be used individually for diagnosing lung tumors

The performance of the two biomarkers was evaluated using receiver operating characteristic (ROC) curve analysis, which is a commonly used method to assess the diagnostic accuracy of biomarkers. The ROC curve plots the true positive rate against the false positive rate at various threshold values, and the area under the curve (AUC) provides a single measure of the diagnostic performance.

**Figure 3:**
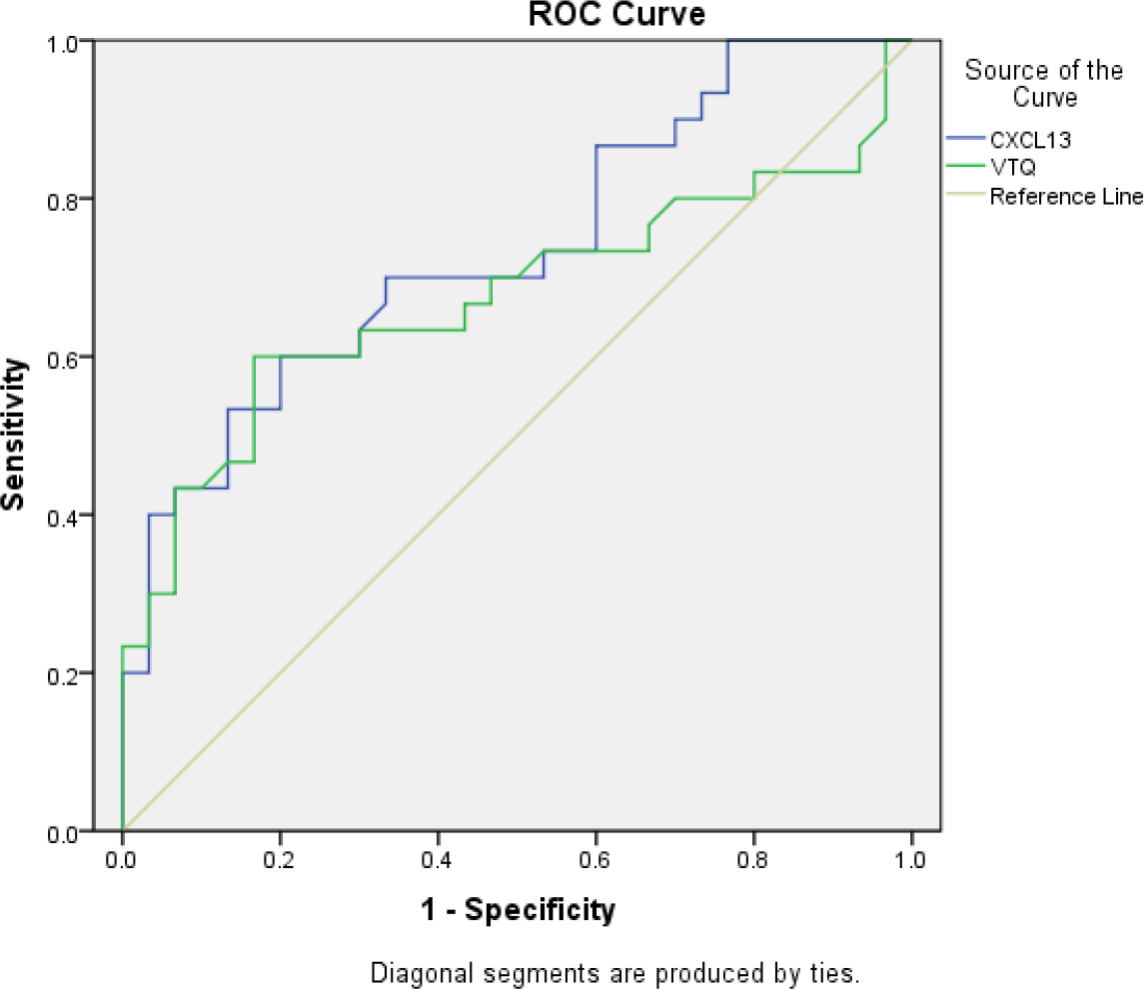
Receiver operating characteristic (ROC) curve analysis of the VTQ result in pleural effusion for diagnosing thoracic tumors. The ROC curve was used to evaluate the diagnostic accuracy of the VTQ result in pleural effusion for diagnosing thoracic tumors. The x-axis represents the false positive rate (1-specificity), and the y-axis represents the true positive rate (sensitivity). The diagonal line represents a random guess. The closer the curve is to the top left corner, the better the diagnostic accuracy. The area under the ROC curve (AUC) was 0.67 (95% confidence interval [CI]: 0.53-0.82). The sensitivity and specificity were 60.0% and 83.3%, respectively, and the optimal diagnostic cutoff was 3.33 m/s. The results indicate that the VTQ result in pleural effusion has a moderate diagnostic accuracy for thoracic tumors.

In this study, the AUC for CXCL13 in diagnosing lung tumors was found to be 0.74 (95% confidence interval [CI]: 0.61-0.86). This indicates that CXCL13 has a good ability to differentiate between patients with lung tumors and those without. The sensitivity and specificity of CXCL13 were 60% and 80%, respectively, which means that 60% of the lung tumor cases were correctly identified, and 80% of the non-tumor cases were correctly identified. The optimal cut-off value, which is the threshold value that provides the best balance between sensitivity and specificity, was found to be 777.82 pg/ml. Similarly, the AUC for VTQ in diagnosing lung tumors was found to be 0.67 (95% CI: 0.53-0.82). This indicates that VTQ also has good diagnostic performance. The sensitivity and specificity of VTQ were 60% and 83.3%, respectively, with an optimal diagnostic cut-off of 3.33 m/s. These results are shown in **Figure 3** and **Table 4**.

**Table 4:** Areas under the ROC curves, sensitivity, specificity, and optimal threshold value for both the two techniques.

	n	AUC and 95%CI	sensitivity	specificity	optimal diagnostic cut-off value	p
CXCL13	60	0.74(0.61, 0.86)	60.0%	80.0%	777.82 pg*/*ml	0.002
VTQ	60	0.67(0.53, 0.82)	60.0%	83.3%	3.33 m*/*s	0.02

The numbers in parenthesis indicate the corresponding 95% confidence interval.

In conclusion, either the VTQ biomarker or the CXCL13 biomarker can serve as an effective individual diagnostic tool for lung tumors.

### Using VTQ and CXCL13 in combination has been demonstrated to have a synergistic effect for diagnosing lung tumors

The study included 60 patients, with 30 cases of lung tumors complicated by malignant pleural effusion in the case group and 30 cases of benign pleural effusion in the control group. The gold standard for diagnosis was based on pathological examination, where malignant cases were coded as 1 and benign cases were coded as 0. Two indicators, VTQ and CXCL13, were measured in each patient and were represented as X1 and X2, respectively (where malignant cases were coded as 1, benign cases were coded as -1, and unknown cases were coded as 0). This is summarized in **Table 5**.

**Table 5:** Results of binary logistic regression using SPSS.

covariate	regression coefficient	standard error	p-value
X1	0.807	0.302	0.008
X2	0.005	0.002	0.002
Constant	6.07	1.698	0.000

The table displays the logistic regression coefficients, standard errors, and p-values for the covariates X1 and X2, obtained using the Binary Logistic procedure in SPSS. The logistic regression equation was used to generate a new variable in the working data table, which contains the predicted probabilities for each individual.

**Figure 4:**
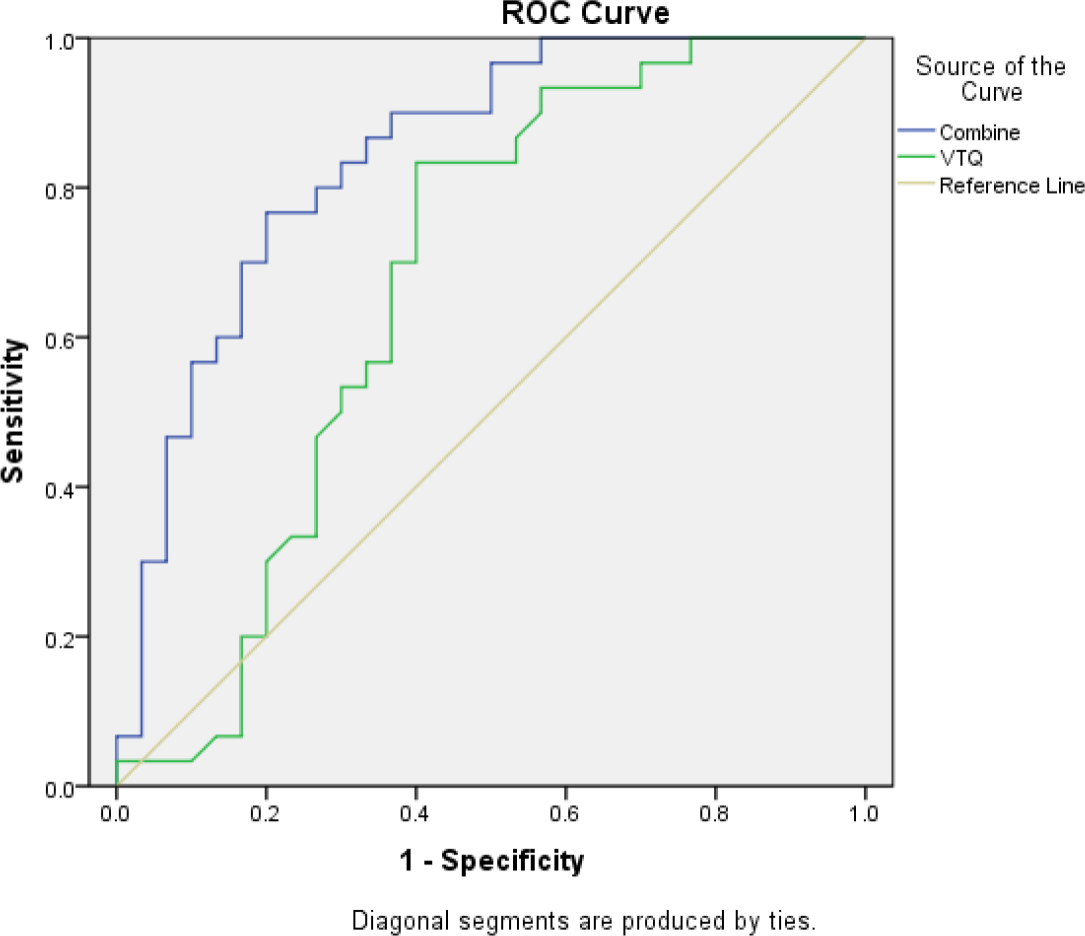
The area under the ROC curve (AUC) for the combined use of CXCL13 and VTQ was 0.842 (0.74, 0.94), which was significantly higher than that of VTQ alone (AUC = 0.674, 95% CI: 0.53, 0.82; P < 0.05), indicating a statistically significant difference between the two methods.

SPSS was used to calculate the individual prediction rate (pre-1) of covariate X1 and the combination of both covariates X1 and X2 (pre-2). The ROC curve analysis was used to evaluate the performance of the variables (pre-1, pre-2) and state variables (group). The results showed that the AUC for covariate X1 (pre-1) in diagnosing malignant pleural effusion was 0.674 (95% confidence interval [CI]: 0.53-0.82). However, the combination of both covariates (pre-2) for the diagnosis of thoracic tumors had a much higher AUC of 0.842 (95% CI: 0.74-0.94). This result is summarised in **Table 6** and **Figure 4**.

**Table 6:** Results of ROC curve analysis using SPSS.

Test variable	AUC and 95% confidence interval	standard error	p-value
Pre-1	0.674(0.53, 0.82)	0.073	0.02
Pre-2	0.842(0.74, 0.94)	0.051	0.00

The table shows the results of ROC curve analysis conducted using the ROC Curve function in the SPSS software. The test variable (TestVariable) was used in conjunction with the diagnosis results of the gold standard as the state variable (StateVariable). The variables Pre-1 and Pre-2 were analyzed to determine the area under the curve (AUC), standard error, p-value, and 95% confidence interval.

In conclusion, the ROC curve analysis using SPSS demonstrated that the combination of VTQ and CXCL13 is a more effective diagnostic tool for lung tumors complicated by malignant pleural effusion compared to the use of either marker alone.

### Posteriori power analysis

A posteriori power analysis was conducted to evaluate the sample size and statistical power of the study. The results of the study showed that both the expression levels of CXCL13 and VTQ were significantly higher in the lung cancer group compared to the control group (P < 0.05), which suggests that the sample size was sufficient to detect a significant difference between the two groups. However, a larger sample size may have increased the statistical power of the study and improved the precision of the results.

The ROC curve analysis of CXCL13 showed an AUC of 0.74 (0.61, 0.86) with an optimal cut-off value of 777.82 pg/ml, indicating that the test has moderate accuracy for diagnosing lung tumors. The ROC curve analysis of VTQ showed an AUC of 0.67 (0.53, 0.82) with a sensitivity of 60.0% and a specificity of 83.3%, which suggests that the test has limited accuracy for diagnosing lung tumors.

The combination of CXCL13 and VTQ for diagnosing thoracic tumors had an AUC of 0.842 (0.74, 0.94), which was significantly higher than either factor alone. This suggests that the combination of the two tests may be a more accurate tool for diagnosing lung tumors compared to using either test alone.

In conclusion, the sample size of the study was sufficient to detect a significant difference between the two groups, but a larger sample size may have increased the statistical power and precision of the results.

## Discussion

Chemokines are a family of small proteins that play a crucial role in various biological processes, including the growth, adhesion, and directional migration of tumor cells. Over the years, an increasing number of studies have highlighted the significant role of chemokines, particularly CXCL13, in tumorigenesis. For instance, CXCL13 has been found to be overexpressed in various solid tumors, such as squamous cell carcinoma and adenocarcinoma, and has been suggested to play a role in regulating the migration and metastasis of cancer cells (2, 3, 5–7).

Studies have shown that the expression level of CXCL13 is positively correlated with the differentiation and stage of lung tumors (2, 3). This has led to the suggestion that CXCL13 may serve as a molecular marker for the diagnosis and prognosis of lung tumors. Although CXCL13 was found to be associated with the differentiation and staging of tumors, it cannot be fully considered as a prognostic indicator without follow-up on changes in CXCL13 levels in pleural effusions before and after treatment and on patient progression-free survival (PFS) and overall survival (OS). In future studies, we will further explore the relationship between changes in CXCL13 levels before and after treatment and overall survival of patients.

To determine the diagnostic value of CXCL13 for lung cancer, the present study established a receiver operating characteristic (ROC) curve, and the area under the curve (AUC) was calculated to be 0.74. Although this suggests that CXCL13 can be used as an independent indicator for lung tumor diagnosis, the study acknowledges that false negative and false positive results may occur.

Alongside traditional diagnostic techniques, such as lung ultrasound, acoustic palpation tissue quantification (VTQ) technology has emerged as a promising tool for evaluating lung tumors. VTQ is based on acoustic radiation force pulse technology, which uses low-pressure pulses to induce local micro-deformation and measure the velocity of shear waves generated by transverse deformation to quantify tissue hardness (8, 15, 16). This study applied VTQ technology to the dynamic and quantitative evaluation of lung tumors and found that the AUC of VTQ for diagnosing lung tumors was 0.67, with the optimal diagnostic cutoff being 3.33 m/s. Although VTQ showed good diagnostic accuracy, the study acknowledges that factors such as thick abdominal fat, breathing problems, and narrow intercostal spaces may affect the accuracy of the measurements (17–19).

To enhance the diagnostic value of VTQ for lung tumors, the present study combined VTQ with CXCL13 evaluation and found that the AUC of the joint diagnosis was significantly higher (0.842(0.74, 0.94))than that of a single indicator. Furthermore, the study determined one or two diagnostic points through linear interpolation and divided positive and negative patients into three categories: positive, suspected positive, and negative. By combining VTQ with CXCL13 evaluation, the study demonstrated that this approach has high diagnostic value in differentiating benign and malignant pleural effusions.

In conclusion, the present study highlights the importance of CXCL13 as a potential molecular marker for the diagnosis and prognosis of lung tumors. Furthermore, the combination of VTQ and CXCL13 evaluation demonstrated high diagnostic value in differentiating benign and malignant pleural effusions. The relative expression of CXCL13 in malignant pleural effusions caused by lung tumors may indicate a poor prognosis, and it is expected to become a new indicator for screening and prognosis of patients with advanced lung cancer. Further studies are necessary to validate these findings and establish the clinical relevance of this diagnostic approach.

## Data availability statement

The original contributions presented in the study are included in the article/supplementary material. Further inquiries can be directed to the corresponding author.

## Ethics statement

The studies involving human participants were reviewed and approved by The Ethics Committee of the Chinese People’s Liberation Army 252 Hospital. The patients/participants provided their written informed consent to participate in this study. Adequate measures were taken to protect the participants’ confidentiality and privacy. Additionally, the study was conducted in accordance with the Declaration of Helsinki and Good Clinical Practice guidelines. The researchers took care to minimize any potential harm to the participants and to ensure that their rights and welfare were protected at all times. It is important to emphasize that the high ethical standards of this study were maintained in order to ensure the validity and reliability of the results, as well as to demonstrate respect for the participants and their contributions to the advancement of scientific knowledge.

## Author contributions

XZ collected and analyzed the data. XZ wrote the original manuscript. KH, QP, and YJ analyzed the data and edited the manuscript. RT supervised the project, reviewed and edited the manuscript. All authors contributed to the article and approved the submitted version.

## References

[1] Xia C Zheng R Zhou M Lin C Zeng H Zhang S. Disparities by province, age, and sex in site-specific cancer burden attributable to 23 potentially modifiable risk factors in china: A comparative risk assessment. Lancet Glob Health (2019) 7:e257–69. doi: 10.1016/S2214-109X(18)30488-1 30683243

[2] Singh R Gupta P Kloecker GH Singh S Lillard JWJ. Expression and clinical significance of cxcr5/cxcl13 in human nonsmall cell lung carcinoma. Int J Oncol (2014) 45:2232–40. doi: 10.3892/ijo.2014.2688 PMC4215579 25271023

[3] Qi X-W Xia S-H Yin Y Jin L-F Pu Y Hua D. Expression features of cxcr5 and its ligand, cxcl13 associated with poor prognosis of advanced colorectal cancer. Eur Rev Med Pharmacol Sci (2014) 18:1916–24. 25010623

[4] Chao C-C Lee W-F Wang S-W chun Chen P Yamamoto A Chang T-M. Cxc chemokine ligand-13 promotes metastasis *via* cxcr5-dependent signaling pathway in non-small cell lung cancer. J Cell Mol Med (2021) 25:9128–40. doi: 10.1111/jcmm.16743 PMC8500967 34427969

[5] Zhu D Ye W Jiang J. Clinical significance of cxcl13/cxcr5 axis in human cancers. Trans Cancer Res (2018) 7:1737–42. doi: 10.21037/tcr.2018.11.26

[6] Hussain M Adah D Tariq M Lu Y Zhang J Liu J. Cxcl13/cxcr5 signaling axis in cancer. Life Sci (2019) 227:175–86. doi: 10.1016/j.lfs.2019.04.053 31026453

[7] Hsieh C Jian C Lin L Low G Ou P Hsu C. Potential role of cxcl13/cxcr5 signaling in immune checkpoint inhibitor treatment in cancer. Cancers (Basel) (2022) 14(2):294. doi: 10.3390/cancers14020294 35053457 PMC8774093

[8] Wei H Lu Y Ji Q Zhou H Zhou X. The application of conventional us and transthoracic ultrasound elastography in evaluating peripheral pulmonary lesions. Exp Ther Med (2018) 16:1203–8. doi: 10.3892/etm.2018.6335 PMC6090271 30116370

[9] Zhi X Wang L Chen J Zheng X Li Y Sun J. Scoring model of convex probe endobronchial ultrasound multimodal imaging in differentiating benign and malignant lung lesions. J Thorac Dis (2020) 12:7645–55. doi: 10.21037/jtd-2020-abpd-005 PMC7797845 33447457

[10] Ozgokce M Yavuz A Akbudak I Durmaz F Uney I Aydin Y. Usability of transthoracic shear wave elastography in differentiation of subpleural solid masses. Ultrasound Q (2018) 34:233–7. doi: 10.1097/RUQ.0000000000000374 30169488

[11] Liu Y Zhen Y Zhang X Gao F Lu X. Application of transthoracic shear wave elastography in evaluating subpleural pulmonary lesions. Eur J Radiol Open (2021) 8:100364. doi: 10.1016/j.ejro.2021.100364 34195303 PMC8233193

[12] Caroli G Dell’Amore A Cassanelli N Dolci G Pipitone E Asadi N. Accuracy of transthoracic ultrasound for the prediction of chest wall infiltration by lung cancer and of lung infiltration by chest wall tumours. Heart Lung Circ (2015) 24:1020–6. doi: 10.1016/j.hlc.2015.03.018 25911140

[13] Lim C-K Chung C-L Lin Y-T Chang C-H Lai Y-C Wang H-C. Transthoracic ultrasound elastography in pulmonary lesions and diseases. Ultrasound Med Biol (2017) 43:145–52. doi: 10.1016/j.ultrasmedbio.2016.08.028 27743728

[14] Sperandeo M Trovato FM Dimitri L Catalano D Simeone A Martines GF. Lung transthoracic ultrasound elastography imaging and guided biopsies of subpleural cancer: A preliminary report. Acta Radiol (2015) 56:798–805. doi: 10.1177/0284185114538424 24951615

[15] Xu J-M Chen Y-J Dang Y-Y Chen M. Association between preoperative us, elastography features and prognostic factors of papillary thyroid cancer with braf(v600e) mutation. Front Endocrinol (Lausanne) (2020) 10:902. doi: 10.3389/fendo.2019.00902 32038479 PMC6987316

[16] Zhong L-C Yang T Gu L-P Ma F. The diagnostic performance of shear wave velocity ratio for the differential diagnosis of benign and malignant breast lesions: Compared with vtq, and mammography. Clin Hemorheol Microcirc (2021) 77:123–31. doi: 10.3233/CH-200813 32924988

[17] Nakano C Nishimura T Tada T Yoshida M Takashima T Aizawa N. Severity of liver fibrosis using shear wave elastography is influenced by hepatic necroinflammation in chronic hepatitis patients, but not in cirrhotic patients. Hepatol Res (2021) 51:436–44. doi: 10.1111/hepr.13617 33462941

[18] Shimada S Kamiyama T Kakisaka T Orimo T Nagatsu A Asahi Y. The impact of elastography with virtual touch quantification of future remnant liver before major hepatectomy. Quant Imag Med Surg (2021) 11:2572–85. doi: 10.21037/qims-20-1073 PMC8107315 34079724

[19] Takuma Y Morimoto Y Takabatake H Tomokuni J Sahara A Matsueda K. Changes in liver and spleen stiffness by virtual touch quantification technique after balloon-occluded retrograde transvenous obliteration of gastric varices and exacerbation of esophageal varices: A preliminary study. Ultraschall Med (2020) 41:157–66. doi: 10.1055/a-0731-0137 30909311

